# Supplementary material for: Field Emission from Carbon Nanotubes on Titanium Nitride-Coated Planar and 3D-Printed Substrates
Source: Nanomaterials (Basel). 2024 Apr 30;14(9):781. doi: 10.3390/nano14090781 (PMC11085237; doi:10.3390/nano14090781)
Supplement: Supplementary file 1 [file nanomaterials-14-00781-s001.zip › nanomaterials-2978546-supplementary.pdf]

Supporting Information

Field Emission from Carbon Nanotubes on Titanium Nitride-Coated Planar and 3D-Printed Substrates

Stefanie Haugg,[1,*] Luis-Felipe Mochalski,[1] Carina Hedrich,[1] Isabel González Díaz-Palacio,[1] Kristian Deneke,[1] Robert Zierold,[1] and Robert H. Blick[1,2]

[1] Center for Hybrid Nanostructures (CHyN), Universität Hamburg, 22761 Hamburg, Germany
[2] Deutsches Elektronen-Synchrotron (DESY), 22607 Hamburg, Germany

* Email corresponding author: shaugg@physnet.uni-hamburg.de

1. EDX measurement of CNTs on TiN/SiN/Si substrate

A line scan using energy-dispersive x-ray spectroscopy (EDX) was performed on the cross section through the carbon nanotube (CNT) layer grown on a titanium nitride (TiN) coated silicon nitride (SiN)/silicon (Si) substrate. The elements were detected as expected for the sample composition distribution and are listed in the legend of Figure S1. The transition from the CNT cross section to the TiN-coated SiN/Si substrate is observed at a point number of about 110 on the vertical axis by the drop of the carbon signal and the simultaneous increase of the signal from the silicon substrate.

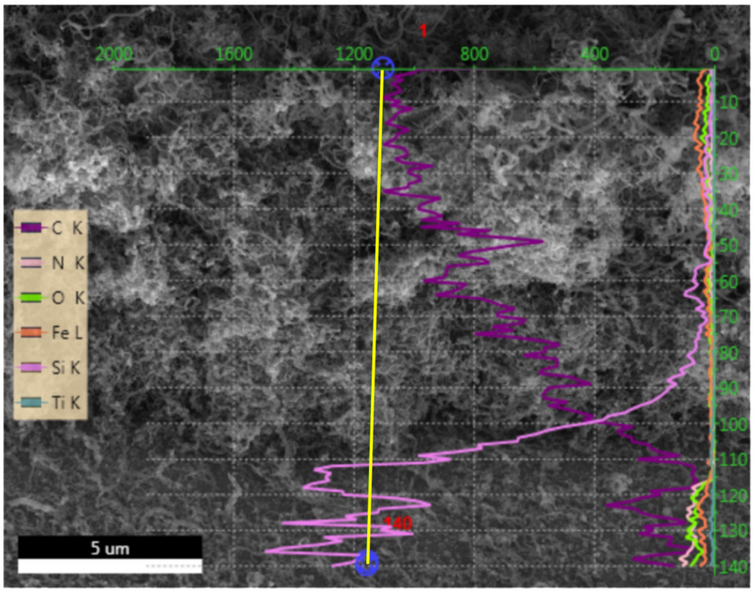

Figure S1. EDX line scan across the CNT layer on a TiN/SiN/Si substrate. The position of the line scan is indicated by the yellow line in the scanning electron microscopy (SEM) image.

2. Murphy-Good (MG) plot of the field emission (FE) data

The MG plot of an individual I-V curve from CNTs on a pristine Si substrate is shown in Figure S2 with $\kappa = 2 - \eta/6 = 1.26685$ ($\eta = 4.3989$ for a work function of 5.0 eV) [1]. The red line indicates the linear section of the FE data that was used to extract the apparent field enhancement factor. For higher voltages—which equals lower values on the left side of the MG plot—a change in slope of the FE data is observed, possibly generated by effects beyond FE. Note, the analysis of all I-V curves was performed by excluding the region of current saturation at higher voltages as shown in Figure S2.

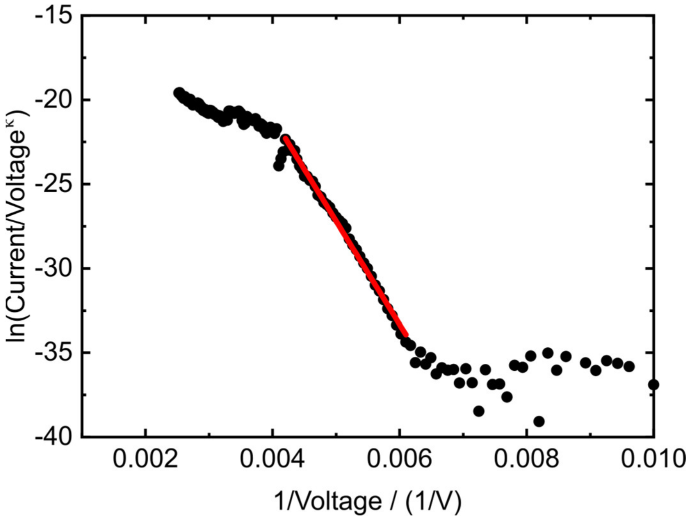

Figure S2. MG plot of an individual I-V curve from CNTs on a pristine Si substrate.

3. Orthodoxy test for the FE data

The orthodoxy test was applied to the FE data from the CNTs on the planar substrates in the MG plot and the results were summarized in Table S1. For the assumed work function of 5.0 eV, the following ranges were used to evaluate the extracted scaled barrier field values (f^{extr}) [2,3]:

"Apparently reasonable" range: $f_{\text{low}} \leq f^{\text{extr}} \leq f_{\text{up}}$ with $f_{\text{low}} = 0.14$ and $f_{\text{up}} = 0.43$

"Clearly unreasonable range" range: $f^{\text{extr}} < f_{\text{lb}}$ or $f^{\text{extr}} > f_{\text{ub}}$ with $f_{\text{lb}} = 0.095$ and $f_{\text{ub}} = 0.71$

Table S1. Orthodoxy test results for the CNTs on the planar substrates.

**Substrate**	$(f_{\text{low}})^{\text{extr}}$	$(f_{\text{up}})^{\text{extr}}$	**Results**
Si	0.13 ± 0.01	0.20 ± 0.03	Inconclusive
TiN on Si	0.14 ± 0.02	0.21 ± 0.03	Pass
SiN	0.09 ± 0.01	0.11 ± 0.01	Fail

TiN on SiN	0.12 ± 0.01	0.17 ± 0.02	Inconclusive
SiN NM	0.12 ± 0.01	0.16 ± 0.02	Inconclusive
TiN on SiN NM	0.15 ± 0.02	0.24 ± 0.05	Pass

4. Stability test for FE from CNTs on 3D-printed structures

Figure S3 shows a stability measurement of the FE current from CNTs on 3D-printed structures measured for an applied electric field of 1.084 V/µm (500 µm emitter-grid distance), which was aborted manually after 20 h. Within the first 6.5 h, the measured current drops by about 45 % to a minimum of 2.7 µA. Subsequently, a stabilization of the emission current is observed around a mean value of (3.7 ± 0.3) µA. Based on these observations, this FE electron source can potentially function for a longer period of time. The observed current fluctuations may be attributed to a combination of emitter changes by resistive heating, mechanical deformation in the electric field, and to interactions with residual gas molecules [4].

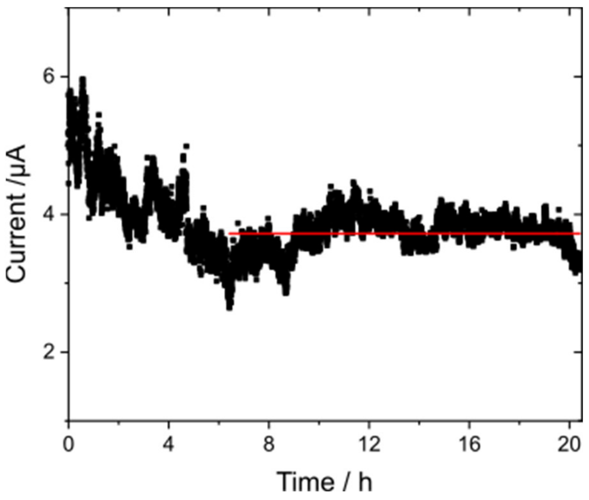

Figure S3. Stability of the FE current from CNTs on 3D-printed structures. The horizontal red line marks the stabilized emission current of (3.7 ± 0.3) µA after 6.5 h. A data point was taken every 10 s until the measurement was aborted manually after 20 h.

5. CNTs on a 3D-printed polymeric structure

Figure S4 shows SEM images of the CNTs on a 9 x 9 array of polymeric cones, which were generated by direct laser writing on a silicon substrate. Prior to the CNT growth, the cones were first covered with 20 nm Al_2O_3 by thermal atomic layer deposition (ALD) and subsequently with 10 nm TiN by plasma-enhanced ALD (PEALD).

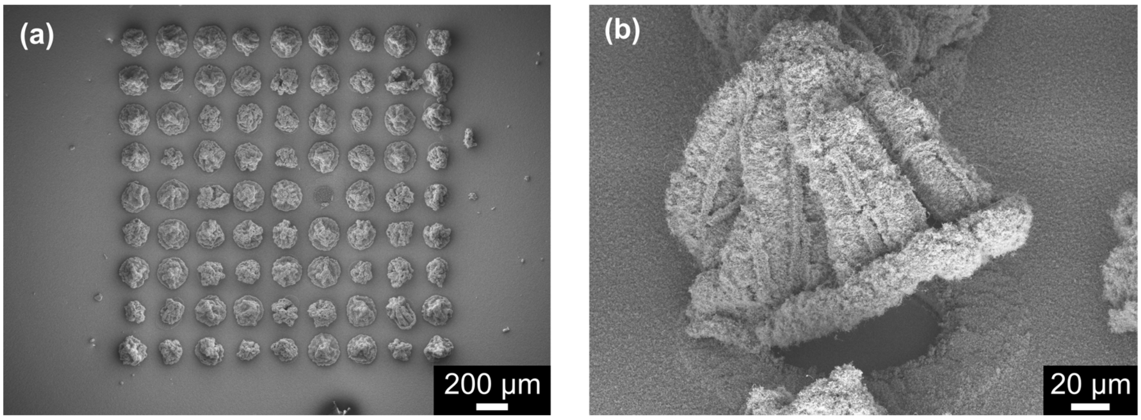

Figure S4. CNTs on 3D-printed polymeric cones. (a) Top view SEM image of the 9 x 9 cone array after CNT growth and FE measurements. (b) Side view of an individual cone that detached from the Si substrate.

6. CNTs on a 3D-printed glass structure

The growth of CNTs was also tested on glass microstructures, which were generated by the same direct laser writing process as the polymeric structures and an additional high-temperature sintering step.

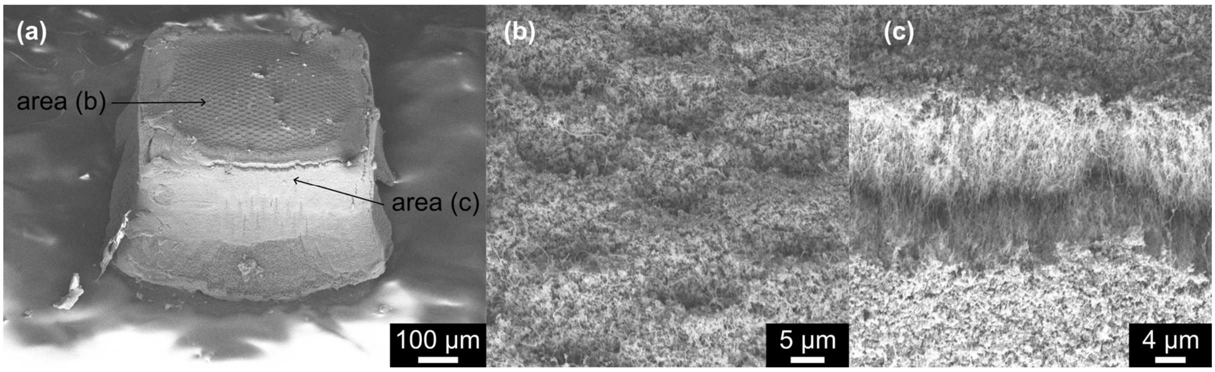

Figure S5. CNTs on a 3D-printed glass structure. (a) The highly entangled network of CNTs that was observed on planar substrates was also found on the glass structure. (b) All sides of

the glass cube were covered by a dense CNT layer, which becomes obvious at the edge of the structure.

7. Full-scan SEM images

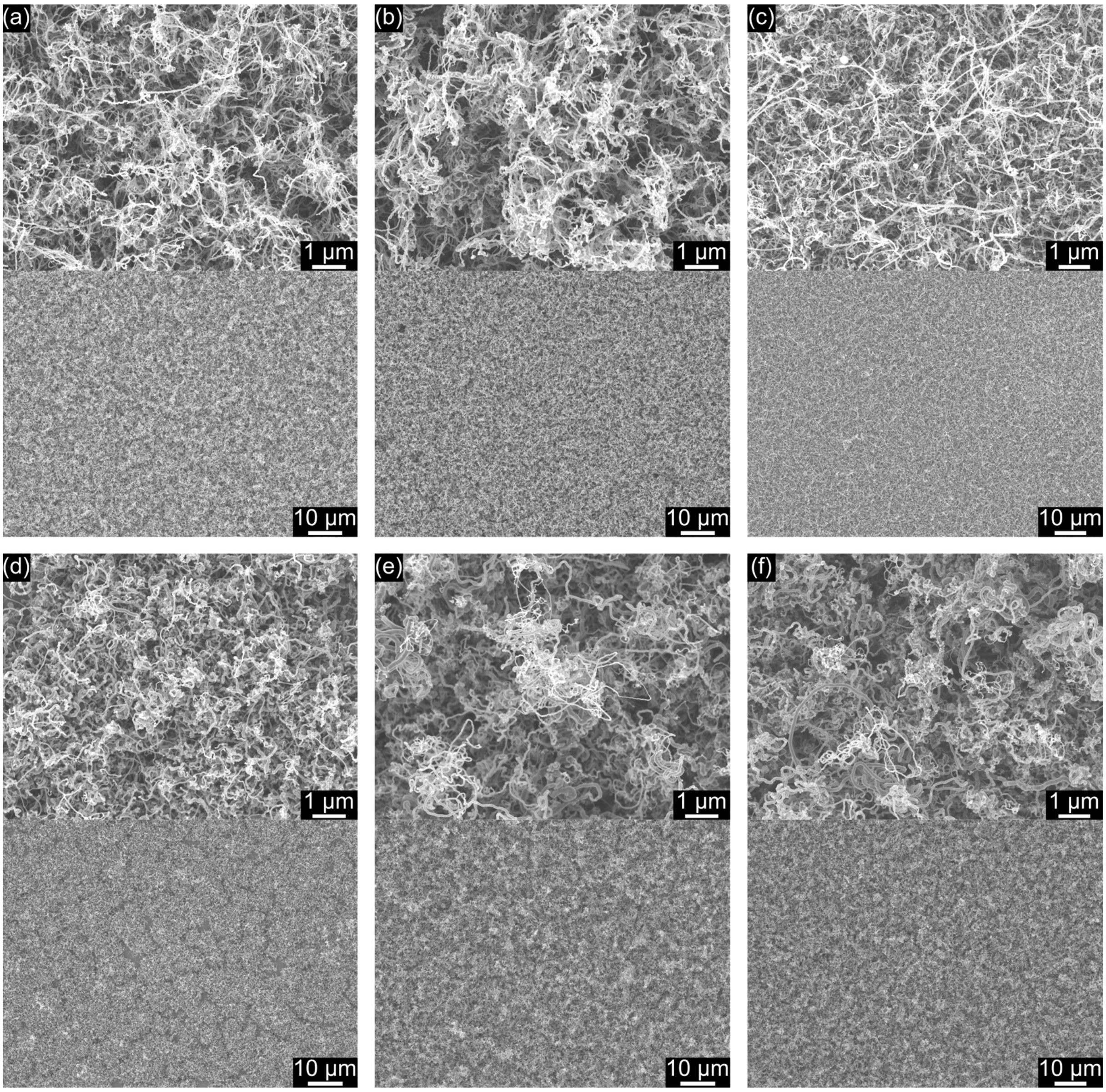

Figure S6. Full-scan SEM images of the cropped SEM images from Figure 1. CNTs on the following pristine substrate types are shown: (a) Si, (b) SiN, and (c) a free-standing SiN NM. (d)–(f): CNTs on the same substrate types coated with an additional TiN film before the CNT growth.

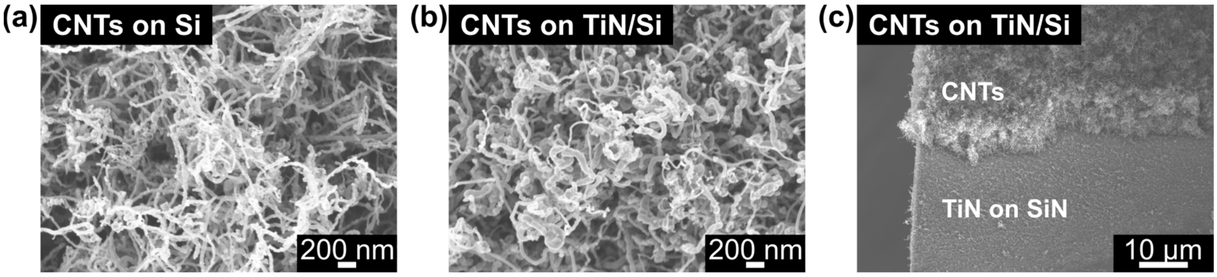

Figure S7. Full-scan SEM images of the cropped SEM images from Figure 2. SEM images of CNTs on (a) pristine Si and on (b) TiN-coated Si. (c) Cross section through the CNT layer on a TiN/SiN substrate.

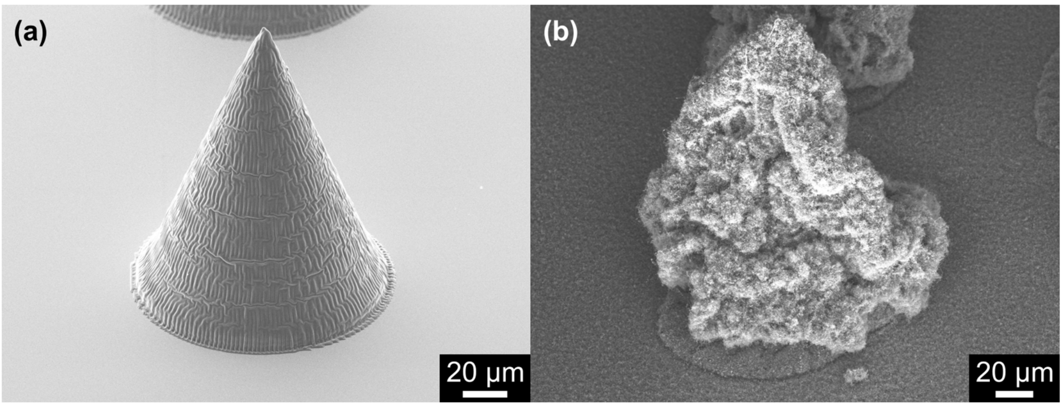

Figure S8. Full-scan SEM images of the cropped SEM images from Figure 6. (a) 3D-printed polymeric cone covered with 10 nm TiN on top of 20 nm Al_2O_3 before CNT growth. (b) CNT layer on an ALD-coated polymeric cone.

References

1. Forbes, R.G. The Murphy-Good plot: A better method of analysing field emission data. *R. Soc. Open Sci.* **2019**, *6*, 190912, doi:10.1098/rsos.190912.

2. Forbes, R.G. Development of a simple quantitative test for lack of field emission orthodoxy. *Proc. R. Soc. A* **2013**, *469*, 20130271, doi:10.1098/rspa.2013.0271.

3. Allaham, M.M.; Forbes, R.G.; Knápek, A.; Mousa, M.S. Implementation of the orthodoxy test as a validity check on experimental field emission data. *J. Electr. Eng.* **2020**, *71*, 37–42, doi:10.2478/jee-2020–0005.

4. Giubileo, F.; Di Bartolomeo, A.; Iemmo, L.; Luongo, G.; Urban, F. Field emission from carbon nanostructures. *Appl. Sci.* **2018**, *8*, 526, doi:10.3390/app8040526.
